# Arbeitsgruppe Interoperabilität: Kerndatensatz und Informationssysteme für Integration und Austausch von Daten in der Medizininformatik-Initiative

**DOI:** 10.1007/s00103-024-03888-4

**Published:** 2024-05-16

**Authors:** Danny Ammon, Maximilian Kurscheidt, Karoline Buckow, Toralf Kirsten, Matthias Löbe, Frank Meineke, Fabian Prasser, Julian Saß, Ulrich Sax, Sebastian Stäubert, Sylvia Thun, Reto Wettstein, Joshua P. Wiedekopf, Judith A. H. Wodke, Martin Boeker, Thomas Ganslandt

**Affiliations:** 1 Datenintegrationszentrum, Universitätsklinikum Jena, Jena, Deutschland; 2 GECKO Institut für Medizin, Informatik und Ökonomie, Hochschule Heilbronn, Heilbronn, Deutschland; 3 TMF – Technologie- und Methodenplattform für die vernetzte medizinische Forschung e. V., Berlin, Deutschland; 4 Institut für Medizinische Informatik, Statistik und Epidemiologie (IMISE), Universität Leipzig, Leipzig, Deutschland; 5 Berliner Institut für Gesundheitsforschung in der Charité – Universitätsmedizin Berlin, Berlin, Deutschland; 6 Institut für Medizinische Informatik, Universitätsmedizin Göttingen, Göttingen, Deutschland; 7 Institut für Medizinische Informatik, Universitätsklinikum Heidelberg, Heidelberg, Deutschland; 8 Institut für Medizinische Informatik & IT Center for Clinical Research, Universität zu Lübeck, Lübeck, Deutschland; 9 Institut für Community Medicine, Medizininformatik, MeDaX, Universitätsmedizin Greifswald, Greifswald, Deutschland; 10 Institut für Künstliche Intelligenz und Informatik in der Medizin, Klinikum rechts der Isar, Technische Universität München, München, Deutschland; 11 Lehrstuhl für Medizinische Informatik, Friedrich-Alexander-Universität Erlangen-Nürnberg, Wetterkreuz 15, 91058 Erlangen, Deutschland

**Keywords:** Sekundärdatennutzung, FHIR, Terminologiedienste, Datenintegrationszentrum, Forschungsdateninfrastruktur, Secondary Use, FHIR, Terminology services, Data integration center, Research data infrastructure

## Abstract

Die Arbeitsgruppe Interoperabilität der Medizininformatik-Initiative (MII) ist die Plattform für die Abstimmung übergreifender Vorgehensweisen, Datenstrukturen und Schnittstellen zwischen den Datenintegrationszentren (DIZ) der Universitätskliniken und nationalen bzw. internationalen Interoperabilitätsgremien. Ziel ist die gemeinsame inhaltliche und technische Ausgestaltung einer über das Forschungsdatenportal für Gesundheit nutzbaren verteilten Infrastruktur zur Sekundärnutzung klinischer Versorgungsdaten. Wichtige Rahmenbedingungen sind dabei Datenschutz und IT-Sicherheit für die Nutzung von Gesundheitsdaten in der biomedizinischen Forschung. Hierfür werden in dezidierten Taskforces geeignete Methoden eingesetzt, um prozessuale, syntaktische und semantische Interoperabilität für Datennutzungsprojekte zu ermöglichen. So wurde der MII-Kerndatensatz, bestehend aus mehreren Modulen mit zugehörigen Informationsmodellen, entwickelt und mittels des Standards HL7® FHIR® implementiert, um fachliche und technische Vorgaben für die interoperable Datenbereitstellung von Versorgungsdaten durch die DIZ zu ermöglichen. Zur näheren Beschreibung dieser Datensätze dienen internationale Terminologien und konsentierte Metadaten. Die Gesamtarchitektur, einschließlich übergreifender Schnittstellen, setzt die methodischen und rechtlichen Anforderungen an eine verteilte Datennutzungsinfrastruktur z. B. durch Bereitstellung pseudonymisierter Daten oder föderierte Analysen um. Mit diesen Ergebnissen der Arbeitsgruppe Interoperabilität stellt die MII eine zukunftsweisende Lösung für den Austausch und die Nutzung von Routinedaten vor, deren Anwendbarkeit über den Zweck der Forschung hinausgeht und eine wesentliche Rolle in der digitalen Transformation des Gesundheitswesens spielen kann.

## Abstract (en)

The interoperability Working Group of the Medical Informatics Initiative (MII) is the platform for the coordination of overarching procedures, data structures, and interfaces between the data integration centers (DIC) of the university hospitals and national and international interoperability committees. The goal is the joint content-related and technical design of a distributed infrastructure for the secondary use of healthcare data that can be used via the Research Data Portal for Health. Important general conditions are data privacy and IT security for the use of health data in biomedical research. To this end, suitable methods are used in dedicated task forces to enable procedural, syntactic, and semantic interoperability for data use projects. The MII core dataset was developed as several modules with corresponding information models and implemented using the HL7® FHIR® standard to enable content-related and technical specifications for the interoperable provision of healthcare data through the DIC. International terminologies and consented metadata are used to describe these data in more detail. The overall architecture, including overarching interfaces, implements the methodological and legal requirements for a distributed data use infrastructure, for example, by providing pseudonymized data or by federated analyses. With these results of the Interoperability Working Group, the MII is presenting a future-oriented solution for the exchange and use of healthcare data, the applicability of which goes beyond the purpose of research and can play an essential role in the digital transformation of the healthcare system.

## Einleitung

Die Arbeitsgruppe Interoperabilität (AG IOP) der Medizininformatik-Initiative (MII) wurde 2017 mit dem Ziel gegründet, eine Abstimmung der geförderten Konsortien zur konkreten technischen Umsetzung der Datenintegrationszentren (DIZ) zu ermöglichen. Hierbei stand insbesondere eine einheitliche standortübergreifende Sekundärnutzung von Versorgungsdaten im Vordergrund. Im Rahmen der kompetitiven Antragstellung waren 4 Konsortien mit jeweils eigenständigen Konzepten für die zu verwendenden Datenstrukturen und Schnittstellen innerhalb der eigenen Standorte vertreten. Die AG der MII war daher essenziell, um die direkte persönliche Interaktion von Beteiligten aller Konsortien, einen offenen Austausch über die jeweiligen Konzepte und die Einigung und Implementierung übergreifender Standards zu ermöglichen. Nur auf diesem Weg konnten innerhalb der föderierten Struktur der MII mit ihrer dezentralen Datenhaltung einheitliche, harmonisierte Vorgehensweisen und technische Infrastrukturen etabliert werden.

Interoperabilität beschreibt „die Fähigkeit von zwei oder mehreren Systemen oder Komponenten, Informationen auszutauschen und die ausgetauschten Informationen weiterzuverwenden“ [1]. Ein Grundsatz der AG IOP sind daher die Auswahl und aktive Weiterentwicklung offener Standards, die unabhängig von einzelnen Softwareprodukten implementiert werden können. Hierbei sind Standardisierungsinitiativen auf nationaler und internationaler Ebene relevant. Ebenso bestand die Notwendigkeit, sich an Umfang und Struktur der an den teilnehmenden Kliniken verfügbaren Datenbestände und den Bedarfen der Use Cases der Konsortien zu orientieren. So wird ein wesentlicher Beitrag zu einer langfristigen Nachnutzung von Arbeitsergebnissen und Daten der MII gemäß den *FAIR Principles *geleistet [2]. Ein weiterer Ansatz der AG IOP ist die Vorgabe iterativer Schritte zur Erreichung der in der MII-Roadmap definierten Meilensteine. Die Zusammenarbeit ist daher grundsätzlich konsortiums- und standortübergreifend und bindet insbesondere bei der Spezifizierung des Kerndatensatzes (KDS) auch interdisziplinäre klinische Expertise ein. Datenschutz und IT-Sicherheit sind entscheidende Aspekte in der MII. Dabei ist nicht nur die technische Umsetzung der Einverständniserklärung der Patient:innen wichtig [3], sondern auch weitere Aspekte wie differierende landesrechtliche Vorgaben, welche einen Einfluss auf die Gestaltung der Gesamtinfrastruktur haben.

Auf Basis dieser Grundprinzipien konnte bereits sehr frühzeitig ein Eckpunktepapier zur Interoperabilität [4] abgestimmt werden, das wesentliche Ziele und Arbeitsprinzipien für die MII definiert und relevante Informationen zur übergreifenden Roadmap und der Umsetzung von Interoperabilitätsmeilensteinen der Aufbau- und Vernetzungsphase der MII enthält. Die AG IOP berücksichtigt aktuelle Entwicklungen von Standards und Werkzeugen aus dem dynamischen Umfeld der Medizininformatik in der allgemeinen Roadmap sowie durch einen jährlichen Release-Zyklus der MII-Gesamtinfrastruktur ab 2024.

Die Etablierung der MII fällt in eine Phase der aktiven Umgestaltung des deutschen Gesundheitswesens in Richtung einer digitalen Transformation der Prozesse zur Generierung und Verwertung von Versorgungsdaten. Eine Vielzahl von Gesetzgebungsverfahren hat hierbei sowohl Rahmenbedingungen für die zunehmende Verfügbarkeit strukturierter Daten als auch ihre Vereinheitlichung auf Basis international akzeptierter Interoperabilitätsstandards und Terminologien geschaffen. Die AG IOP ist hierbei sowohl inhaltlich als auch mit ihren agierenden Personen in Gremien außerhalb der MII (z. B. dem Interop Council [5]) eng eingebunden. Dies stellt den Austausch sowie die Berücksichtigung von Anforderungen der wissenschaftlichen Sekundärnutzung von Routinedaten sicher. So konnte durch die Pilotierung der Terminologie SNOMED CT [6] in der MII die Grundlage für die Etablierung einer Nationallizenz geschaffen werden [7].

Ziel dieses Artikels ist es, die wesentlichen Arbeitsgrundsätze und verwendeten Methoden der AG IOP sowie die in der bisherigen Laufzeit erzielten Ergebnisse darzustellen. Aufgrund der Breite des Arbeitsspektrums können in diesem Artikel nur Schlaglichter hervorgehoben werden.

## Methoden der AG Interoperabilität

### Übergreifende Arbeitsweise der AG IOP und ihrer Taskforces

Die AG IOP setzte sich in den ersten 2 Förderphasen der MII aus benannten Mitgliedern der 4 MII-Konsortien zusammen, seit der aktuellen Ausbau- und Erweiterungsphase wurde sie um Vertreter:innen der Digitalen Fortschrittshubs und konsortiumsübergreifenden Use Cases ergänzt. Für die Detailarbeit in mehreren Iterationen wurden Taskforces (TF) eingerichtet, die aus Mitgliedern der AG IOP und weiteren Fachexpert:innen bestehen:

- TF Consent Umsetzung (Beteiligung der AGs Consent und Data Sharing – DaSh),
- TF Datenselektion, Datenbereitstellung und Testdaten (DDT),
- TF Kerndatensatz (KDS),
- TF Metadaten,
- TF Prozessmodelle (Beteiligung der AG DaSh),
- TF Terminologiedienste (TD; Beteiligung der AG DaSh),
- TF Übergreifende Schnittstellen (ÜS; Beteiligung der AG DaSh),
- TF Verteilte Analysen (VA; Beteiligung der AG DaSh).

Die TFs sind interdisziplinär gemäß den jeweiligen Themengebieten besetzt und arbeiten eigenständig zum Teil in Teams Anforderungen und Lösungsansätze aus. In der AG IOP werden Konzepte der TFs vorgestellt und übergreifend abgestimmt. Neben den Sitzungen werden auch Umlaufverfahren angewendet. Ergebnisse, für die eine bindende Umsetzung durch die MII-Konsortien erforderlich ist, werden als Beschlussvorlagen im Nationalen Steuerungsgremium behandelt.

Eine Basis für Arbeiten der AG IOP sind die entwickelten Standards und technischen Beschreibungen des Joint Initiative Council (JIC) wie etwa die International Patient Summary (IPS; [8]). Das JIC ist ein internationaler Zusammenschluss von Standardisierungsorganisationen wie HL7 (Health Level Seven International), IHE (Integrating the Healthcare Enterprise), CDISC (Clinical Data Interchange Standards Consortium) etc. Er setzt sich für die Harmonisierung von Gesundheitsdaten ein und kooperiert mit der Weltgesundheitsorganisation (WHO).

In der MII werden für Planung, Spezifikation und Dokumentation verschiedene Methoden eingesetzt wie Entity-Relationship(ER)-Modelle [9], Klassendiagramme der Unified Modelling Language (UML; [10]) für Informationsmodelle, Business Process Model and Notation (BPMN) 2.0 [11] für Prozessmodelle sowie die 3‑Ebenen-Methodik „Three-layer Graph-based Meta Model“ (3LGM^2^; [12]) für die Gesamtarchitektur.

Die Entwickler-Plattform GitHub dient als Plattform für die übergreifende Zusammenarbeit, u. a. als Projektmanagement-Werkzeug sowie als Softwareversionsverwaltung mit öffentlichen Repositorien [13].

### Steuerung und Entwicklung der Kerndatensatz-Module

**Abb. 1:**
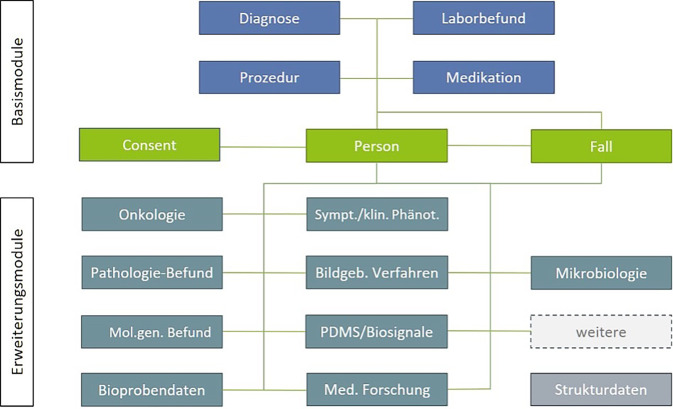
Module des MII-Kerndatensatzes Ende 2023 (*blau*: medizinische Inhalte, *grün*: nichtmedizinische Inhalte, *dunkelgrün/grau*: Erweiterungsmodule; Medizininformatik-Initiative [52]). *klin. Phänot.* klinischer Phänotyp, *Med.* Medizinische, *Mol.gen.* molekulargenetischer, *PDMS* Patientendatenmanagementsysteme, *Sympt.* Symptome; © Medizininformatik-Initiative 2023 [52]

Als Kerndatensatz (KDS) wird in der MII die konkrete Sammlung von Versorgungsdaten in den DIZ bezeichnet. Darüber hinaus umfasst der Begriff auch die in der MII entwickelten Informationsmodelle und technischen Umsetzungen, wie diese Daten strukturiert werden und wie auf sie zugegriffen werden kann [14]. Bereits in der Konzeptphase 2016–2017 wurde von Mitgliedern der AG IOP die Vielfalt der unterschiedlichen Behandlungsdaten nach den Kriterien digitaler Verfügbarkeit, Strukturiertheit und Notwendigkeit für den Zweck der Sekundärnutzung untergliedert und priorisiert [15]. Für die meisten Fragestellungen erforderliche Datenelemente wie der Personen- und Fallzusammenhang, Diagnosen und Prozeduren wurden als *Basismodule* deklariert, die jedes DIZ im gleichen interoperablen Format bereitstellen muss. Darüber hinausgehende fachspezifische und projektspezifische Daten werden in *Erweiterungsmodulen* abgebildet, die initial nur von teilnehmenden DIZ umgesetzt werden (Abb. 1).

Für die technische Spezifikation der KDS-Module hat die AG IOP im April 2019 im Rahmen eines konsortienübergreifenden Einigungsprozesses den für Behandlungsdaten bedeutsamen Standard FHIR® R4 (Fast Healthcare Interoperability Resources Release 4) von HL7 [16] gewählt und ab 2020 auf Basis einer veröffentlichten Governance [17] nach den Regeln von HL7 Deutschland ballotiert [18].

**Abb. 2:**
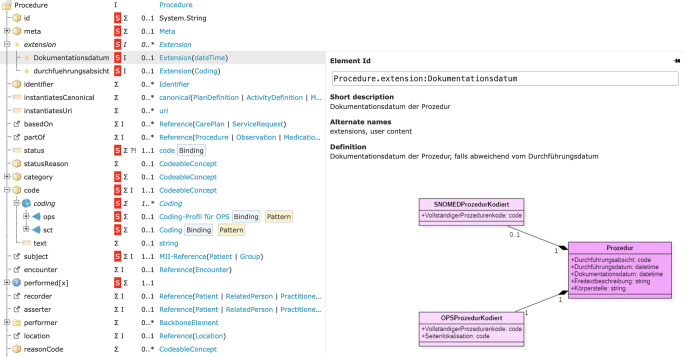
Ausschnitt aus dem UML-Diagramm (*rechts unten*) sowie aus dem FHIR-Profil (*links* und *oben*) für das Kerndatensatzmodul Prozedur mit einer Erweiterung für das Dokumentationsdatum; HL7 Deutschland e. V. [18]; © Medizininformatik-Initiative 2023 [52]

KDS-Module entstehen durch iterative Anpassung des internationalen FHIR-Standards an die Gegebenheiten des deutschen Gesundheitswesens und an den Anwendungszweck der Sekundärnutzung von Routinedaten (sog. Profilierung). Auf Basis dessen wird zunächst ein Informationsmodell mit dem Werkzeug ART-DECOR [19] bzw. mit UML erstellt und freigegeben. Die Erstellung der Profile erfolgt mithilfe von FHIR Shorthand [20]. Die Module und ihre Umsetzung werden in technischen Leitfäden, sog. Implementation Guides (IG), beschrieben, die mit den Werkzeugen Simplifier [21] und HL7 IG Publisher [22] erstellt werden (Abb. 2). Neben der kontinuierlichen Weiterentwicklung der KDS-Module wurden 2023 als zusätzliches Format einwöchige Workshops für die Initiierung neuer Module etabliert, bei denen ein neu etabliertes Team zunächst in Methodik und Werkzeugen für die Anforderungserhebung und Profilierung geschult wird und anschließend gemeinsam den ersten Prototyp eines neuen Moduls erarbeitet.

In HL7 FHIR spielen kodierte Datenelemente mit Verknüpfungen zu Konzepten aus medizinischen Terminologien zur Sicherstellung der semantischen Interoperabilität eine tragende Rolle [23–26]. Beispielsweise stellt HL7 FHIR zur Kommunikation von z. B. Laborwerten die Observation-Ressource bereit. Welcher konkrete Laborwert in einer Observation wiedergegeben wird, muss allgemein verständlich kodiert werden. Dies spiegelt sich in der Profilierung durch die Notwendigkeit wider, Wertelisten relevanter Codes (*Value Sets*) zu definieren und verpflichtend zu hinterlegen. Hierzu werden etablierte semantische Standards und Terminologien verwendet. Weiterhin besteht die Notwendigkeit, parallele Kodierungen zu erlauben, um ein Datum (z. B. Diagnose einer Seltenen Erkrankung) auf mehrere Terminologien (z. B. ICD-10-GM, ORPHAcodes und SNOMED CT) abbilden zu können. Einen Überblick über verwendete Terminologien (und gängige Abkürzungen) der KDS-Module liefert Tab. 1.

**Tab. 1:** Terminologien, auf die der Kerndatensatz (*KDS*) der Medizininformatik-Initiative (*MII*) verweist (Stand April 2024)

Terminologie/Klassifikation	Nutzende Basismodule	Nutzende Erweiterungsmodule
SNOMED CT, eine Referenzterminologie zur Darstellung komplexer medizinischer Sachverhalte	Person, Diagnose, Prozedur, Medikation, Laborbefund	Pathologie, molekulargenetischer Befundbericht, Bioproben, Bildgebung, Intensivmedizin, Mikrobiologie
LOINC (Logical Observation Identifiers Names and Codes), eine Terminologie für u. a. Laboruntersuchungen	Person, Laborbefund, Consent	Pathologie, Intensivmedizin, Mikrobiologie
UCUM (Unified Code for Units of Measure), eine Kodierrichtlinie für maschinenlesbare Einheitensymbole	Laborbefund, Medikation	Pathologie, molekulargenetischer Befundbericht, Bioproben, Intensivmedizin, Mikrobiologie
ICD-10-GM (International Statistical Classification of Diseases and Related Health Problems, 10th Revision, German Modification)	Diagnose	Molekulargenetischer Befundbericht
Alpha-ID, das alphabetische Verzeichnis der ICD-10-GM mit assoziierten Identifier-Codes, mit hoher Relevanz für die Kodierung seltener Erkrankungen	Diagnose	Molekulargenetischer Befundbericht
ORPHAcodes (Orphanet Nomenclature of Rare Diseases) zur Kodierung seltener Erkrankungen	Diagnose	Molekulargenetischer Befundbericht
ICD-O‑3 (International Classification of Diseases for Oncology)	Diagnose	Bioproben
OPS (Operations- und Prozedurenschlüssel)	Prozedur	–
HGNC (Hugo Gene Nomenclature Committee-Nomenklatur) zur Benennung von Genloci	–	Molekulargenetischer Befundbericht
ATC (Anatomic Therapeutic Chemical Classification System)	Medikation (deutsche Version und WHO)	–
EDQM Standard Terms (European Directorate for the Quality of Medicines & HealthCare) zur Benennung von Begriffen in Arzneimittel-Beipackzetteln	Medikation	–
ASK (Arzneistoffkatalog) UNII (Unique Ingredient Identifier) CAS (Chemical Abstracts Service) Systeme zur Kodierung von Wirkstoffen in Arzneimitteln	Medikation	–
IHE XDS (Integrating the Healthcare Enterprise – Cross-Document Sharing) *Fallkontext bei Dokumentenerstellung*	Medikation	–
HL7 FHIR/HL7 V3/HL7 V2: *diverse Terminologien*	Person, Diagnose, Prozedur, Fall, Laborbefund, Medikation	Pathologie, molekulargenetischer Befundbericht, Bildgebung, Mikrobiologie, Strukturdaten, Bioproben
HL7 Deutschland e. V. (Technisches Komitee FHIR): *diverse Terminologien*	Person, Fall	Strukturdaten
ISO/IEEE 11073-10101 (Standard zur Gerätekommunikation am Point of Care)	–	Intensivmedizin
DICOM (Standard zur Kommunikation von Bildgebungsdaten)	–	Bildgebung
*Selbstdefiniert*	Person (Vitalstatus), Consent (Policy)	Mikrobiologie (gemäß mikrobiologischen Guidelines), Strukturdaten (Organisationstyp)
*Sonstige*	–	Biobank (BBMRI), Mikrobiologie (Pathogene)

Die Auswahl der Terminologien erfolgte im Zuge der Informationsmodellierung durch die Kerndatensatzteams unter Berücksichtigung der in der Versorgungsroutine erfassten Daten

Innerhalb des Governance-Konzepts wird zunächst das im jeweiligen Modulteam abgestimmte Informationsmodell und anschließend der IG durch die AG IOP fachlich und das Nationale Steuerungsgremium (NSG) organisatorisch freigegeben. Abschließend erfolgt die Einbindung der Community im Rahmen eines öffentlichen Ballotierungsprozesses, bei dem die praktische Nutzbarkeit der Spezifikation im Vordergrund steht.

Nach der erfolgreichen Ballotierung von KDS-Modulen ermöglichen die DIZ die Bereitstellung von Behandlungsdaten aus klinischen Primärdokumentationssystemen im interoperablen Format des KDS durch ETL(Extract–Transform–Load)-Verfahren (siehe auch Beitrag von Albashiti et al. in diesem Themenheft). Nur durch die Überführung verteilter, herstellerspezifisch gespeicherter Versorgungsdaten in das KDS-Format werden übergreifende Machbarkeitsanfragen, Datenselektion sowie -bereitstellung bzw. verteilte Analysen mithilfe der MII-Gesamtinfrastruktur möglich.

### Entwicklungsprozess und übergreifende Release-Planung

Der Aufbau der Gesamtinfrastruktur erfolgte mithilfe von Projectathons, anhand derer zunächst die Anwendbarkeit erarbeiteter KDS-Spezifikationen, später die Funktionsfähigkeit einzelner Infrastrukturkomponenten und zuletzt das organisatorische und technische Zusammenspiel aller Komponenten und übergreifender Prozesse erprobt werden konnten.

Mit zunehmender Reife der Gesamtinfrastruktur erfolgte der Aufbau einer Testinfrastruktur, die sich aus zentralen und verteilten Systemen zusammensetzt. Die Korrektheit und Anwendbarkeit der Entwicklungen, z. B. Schnittstellen, werden vor ihrer Inbetriebnahme über die verteilte Testinfrastruktur geprüft und in Anbindungs-Hackathons gemeinsam mit der Community erprobt.

Um die Entwicklung der Komponenten der Gesamtinfrastruktur aufeinander abzustimmen und Umsetzungen insbesondere für die DIZ planbar halten zu können, wurde eine übergreifende Release-Planung abgestimmt. Der Prozess umfasst:

- die Ermittlung und Priorisierung von Anforderungen der MII-Teilprojekte,
- die Erstellung und Abstimmung von Spezifikationen,
- die technische Umsetzung und Erprobung,
- das Ausrollen der Funktionen,
- den Abschluss des Release-Wechsels.

## Ergebnisse der AG Interoperabilität

### MII-KDS

Verabschiedete KDS-Module liegen als IG veröffentlicht vor, zusätzlich werden die erzeugten FHIR-Profile in Form von Packages angeboten, die u. a. DIZ zur Validierung ihrer ETL-erzeugten Datensätze nutzen. So wird z. B. im IG des Moduls Medikation [22, 23] beschrieben, wie die Angabe einer Medikamentenpackung mit Pharmazentralnummer erfolgt, sodass in Deutschland gebräuchliche Medikationen für die Sekundärnutzung in der Forschung erschließbar werden. Zusätzlich kommen internationale Terminologien wie LOINC (Logical Observation Identifiers Names and Codes) für Laboruntersuchungen oder SNOMED CT [7] zum Einsatz, etwa für die Klassifikation von Erregern im Erweiterungsmodul Mikrobiologie [24] oder für die Angabe von Verwandtschaftsverhältnissen im molekulargenetischen Befundbericht [27].

Diese Profilierungsarbeit muss bestehende oder parallel erarbeitete Spezifikationen berücksichtigen, um Interoperabilität zu gewährleisten. So basieren die KDS-Module z. B. auf Vorarbeiten aus der IPS [5] und aus den Basisprofilen von HL7 Deutschland [28]. Gleichzeitig haben die Teams der KDS-Module Abstimmungen mit der Gematik (ISiK-Leitfäden [Informationssysteme im Krankenhaus]^1^) und der Kassenärztlichen Bundesvereinigung bzw. mio42 (MIO [Medizinische Informationsobjekte]^2^). Im Ergebnis sind viele dieser Spezifikationen miteinander kompatibel und entsprechende Daten lassen sich in die jeweiligen Profile überführen, bspw. das ISiK-Patient:innenprofil in das MII-Patient:innenprofil [29].

Aufgrund der Bedarfe aus vielen neuen MII-Teilprojekten der Ausbau- und Erweiterungsphase sowie aus weiteren deutschen Forschungsinitiativen werden die Spezifikationen des KDS auf Basis der o. g. Release-Planung kontinuierlich erweitert. So stehen in nächster Zeit Spezifikationsaufgaben zur Kardiologie, zu molekularen Tumorboards, zu Mental Health und zu Patient-reported Outcome Measures (PROMs) an. Aufgrund der Vorreiterrolle der MII bei diesen Arbeiten eignet sich der KDS zur Evaluation des Einsatzes von FHIR-Profilen in der Gesundheitsversorgung. Zukünftige Arbeiten im Bereich FHIR-Interoperabilität in Deutschland können so auf den Ergebnissen der MII, ISiK und MIO aufbauen.

Eine Übersicht über die einzelnen Module des KDS, ihre Auffindbarkeit im Web sowie Beispielprojekte, in denen sie jeweils zur Anwendung kommen, gibt Tab. 2. Der aktuelle Arbeitsstand der Module sowie Links zu den Implementierungsleitfäden und Downloads der Packages sind im KDS-Wiki verfügbar [30]. Ihr Nutzungsstand ist über das Transparenzregister mit der Übersicht durchgeführter Projekte im Forschungsdatenportal für Gesundheit ersichtlich (Tab. 2).

**Tab. 2:** Übersicht über Arbeits- und Nutzungsstand der Kerndatensatzmodule

Modul	Nutzungsbeispiele
**Veröffentlichte Basismodule:** https://www.medizininformatik-initiative.de/de/basismodule-des-kerndatensatzes-der-mii	
Person [50]	*Alle Projekte*
Fall [50]	*Alle Projekte*
Consent [3]	Projekt MII-VHF: https://forschen-fuer-gesundheit.de/projekt1.php
Diagnose [50]	*Nahezu alle Projekte*
Prozedur [50]	*Nahezu alle Projekte*
Laborbefund [51]	Projekt DiaClusT: https://forschen-fuer-gesundheit.de/projekt_diaclust.php
Medikation [25, 26]	Projekt INTERPOLAR: https://www.gesundheitsforschung-bmbf.de/de/interpolar-medizininformatik-use-case-interventional-polypharmacy-drug-interactions-risks-16151.php
**Erweiterungsmodule:** https://www.medizininformatik-initiative.de/de/erweiterungsmodule-des-kerndatensatzes-der-mii	
Pathologiebefund	Projekt PM4Onco: https://www.gesundheitsforschung-bmbf.de/de/pm4onco-daten-besser-nutzbar-machen-krebserkrankungen-wirkungsvoller-behandeln-16626.php
Molekulargenetischer Befundbericht [27]	Projekt PM4Onco: https://www.gesundheitsforschung-bmbf.de/de/pm4onco-daten-besser-nutzbar-machen-krebserkrankungen-wirkungsvoller-behandeln-16626.php
Intensivmedizin	Projekt ACRIBiS: https://www.gesundheitsforschung-bmbf.de/de/acribis-medizininformatik-use-case-verbesserung-der-kardiovaskularen-risikoidentifizierung-16509.php
Bioprobendaten	Projekt ABIDE_MI: https://www.gesundheitsforschung-bmbf.de/de/konsortien-ubergreifender-use-case-abide-mi-biobanken-und-datenintegrationszentren-13716.php
Mikrobiologie [24]	Projekt RISK PRINCIPE: https://www.gesundheitsforschung-bmbf.de/de/risk-principe-medizininformatik-use-case-risk-prediction-for-risk-stratified-infection-16934.php
Symptom/klinischer Phänotyp	Projekt GeMTeX: https://www.gesundheitsforschung-bmbf.de/de/gemtex-medizininformatik-plattform-german-medical-text-corpus-16820.php
Onkologie	Projekt PM4Onco: https://www.gesundheitsforschung-bmbf.de/de/pm4onco-daten-besser-nutzbar-machen-krebserkrankungen-wirkungsvoller-behandeln-16626.php
Medizinische Forschungsvorhaben	*Alle Projekte*
Befunde bildgebender Verfahren	Projekt OMI: https://www.gesundheitsforschung-bmbf.de/de/omi-medizininformatik-plattform-open-medical-inference-16916.php
Strukturdaten	*Alle Projekte*

### Terminologiedienste

Für Auswahl, Erweiterung und technische Integration von in der MII relevanten Codesystemen und Value Sets im KDS sowie in den Use Cases wurde die TF TD gegründet. Ein wichtiges Arbeitsergebnis war die Definition von einheitlichen Richtlinien zur Namensvergabe mit Verbindlichkeit für die Weiterentwicklung der KDS-Module und der KDS-Governance.

Die konsequente Etablierung der Nutzung terminologischer Dienste in der MII dient zur Sicherstellung semantischer Interoperabilität, z. B. durch die Zuordnung standortspezifischer Begriffslisten (z. B. Laborcodes) der Primärdokumentation zu entsprechenden Value Sets internationaler Terminologien (z. B. LOINC) während der ETL-Prozesse in den DIZ. Dazu wurde ein Anforderungskatalog für Terminologieserver erarbeitet, der auf einer im Jahr 2022 durchgeführten Umfrage zu Terminologien und Klassifikationen unter den DIZ basiert.

Seit Anfang 2023 wird ein solcher zentraler Terminologieserver durch das MII-Projekt „SU-TermServ“ [31] etabliert, der Terminologien und Value-Set-Ressourcen für die dezentrale Verarbeitung abrufbar bereitstellt. Weiterhin wurde die Etablierung von SNOMED CT als Referenzterminologie [7] innerhalb der KDS-Module vorangetrieben (Tab. 2).

### Metadaten

Die übergreifende Definition und Verwertung von harmonisierten Metadaten zur Datenverfügbarkeit, Datenprovenienz und Datenqualität ist ein Ziel, an dem die TF Metadaten gemeinsam mit anderen arbeitet. Die Anreicherung der klinischen Daten mit Metadaten ermöglicht es u. a., den Kontext der Erhebung (in heterogenen Primärdokumentationssystemen) und die Verarbeitungsschritte strukturiert abzurufen.

Für weitere Metadaten wie aggregierte Daten, Beschreibung von Datensätzen, Schnittstellen oder Softwaresystemen wurden Empfehlungen erarbeitet, welche die Verfügbarkeit und Inhalte von Datenexporten an den DIZ adressieren [32]. Dabei wurden auch internationale Standards außerhalb der Gesundheitsversorgung beachtet, die für eine Einbettung in Initiativen wie die European Open Science Cloud oder den European Health Data Space (EHDS) wichtig sind, bspw. DataCite und DCAT-AP (Data Catalog – Vocabulary Application Profile; [33]).

Im Rahmen der Projectathons wurden außerdem praxisnahe Frameworks wie das in Kohortenstudien bewährte Werkzeug dataquieR [34] eingesetzt, um Untersuchungen zur Datenqualität zu ermöglichen, d. h. zur syntaktischen und inhaltlichen Konsistenz und Plausibilität bzw. zur Auszeichnung und Interpretation von Fehlerwerten.

### Gesamtarchitektur und übergreifende Schnittstellen

Im Rahmen der AG DaSh (siehe auch Beitrag von Kirsten et al. in diesem Themenheft) wurden die rechtlichen und organisatorischen Rahmenbedingungen für den Datenzugang und die Datennutzung in der MII entwickelt. Diese umfassen insbesondere die Definition der übergreifenden Prozesse für Machbarkeitsanfragen, das Antrags- und Vertragsmanagement sowie die daraus resultierenden Datennutzungsprojekte. Diese Prozesse werden durch eine verteilte Infrastruktur umgesetzt, welche aufgrund der standortspezifischen und konsortialen Strukturen eine große Heterogenität an Systemen in DIZ (z. B. ETL-Strecken, FHIR-Server) und in zentralen Diensten (z. B. Pseudonymisierungsdienste, Datenmanagementstellen [DMSt]) aufweist. Daher werden in der AG IOP die o. g. offenen Standards eingesetzt, um übergreifende und interoperable Schnittstellen zu definieren, welche automatisierte Prozesse ermöglichen.

**Abb. 3:**
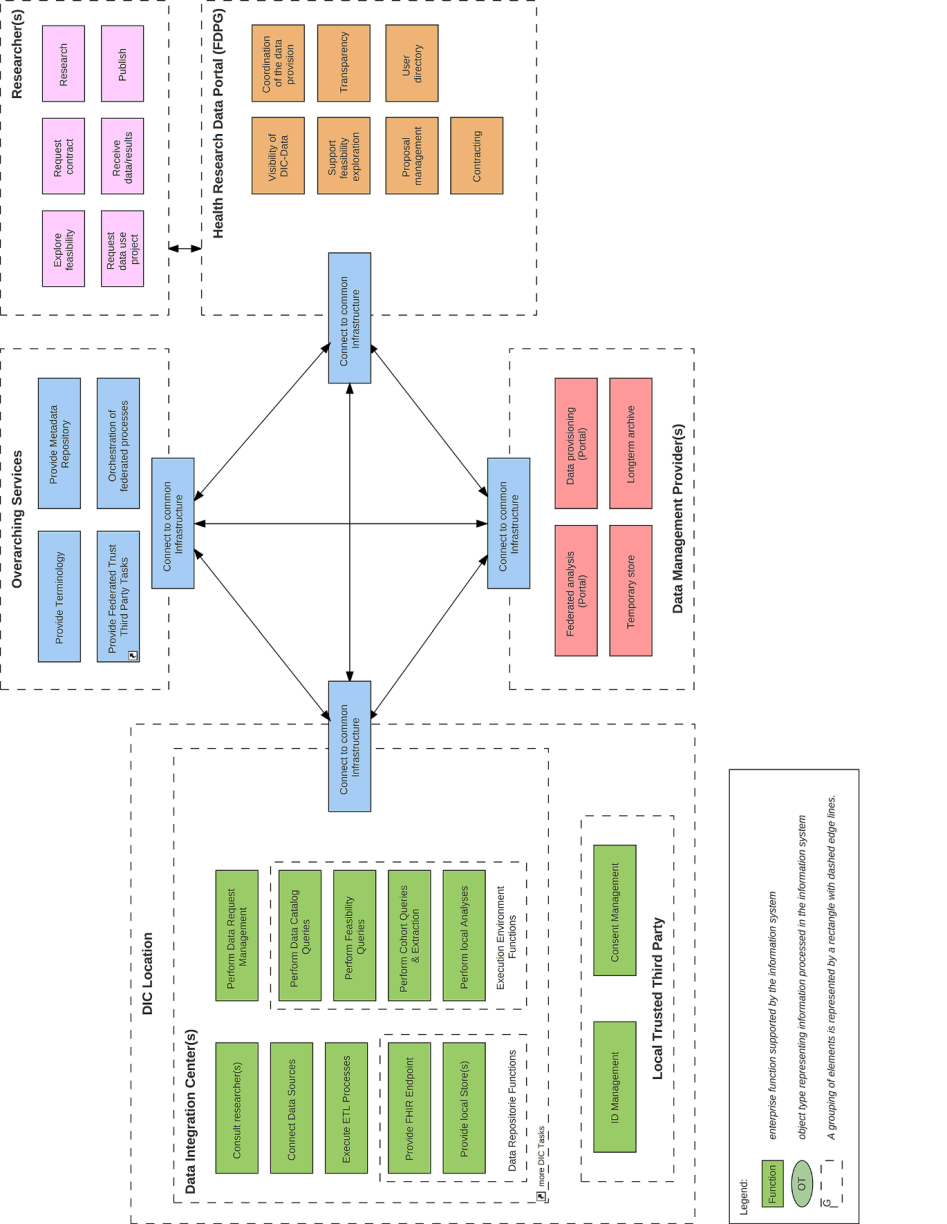
Fachliche Ebene des 3LGM-Gesamtarchitekturmodells der MII: Datenintegrationszentren (*grün*), Datenmanagementstellen (*rot*), Forschungsdatenportal für Gesundheit (*orange*), Forscher:innen (*lila*) sowie gemeinsam genutzte Dienste (*blau*), die miteinander durch die verteilte Infrastruktur verbunden sind; Medizininformatik-Initiative [35]; © Medizininformatik-Initiative 2023 [52]

Um die Komplexität der übergreifenden Schnittstellen zu analysieren, wurde ein 3LGM^2^-Gesamtarchitekturmodell entwickelt (Abb. 3), das sowohl eine fachliche Modellebene mit den Hauptakteuren – Forscher:innen, DIZ, DMSt, FDPG und übergreifenden Diensten – als auch eine logische Modellebene mit den technischen Schnittstellen und Softwarekomponenten abbildet [35]. Zur technischen Kommunikation zwischen den Hauptakteuren wurden z. B. die Middleware-Plattformen Data Sharing Framework (DSF; [36, 37]) und AKTIN (Aktionsbündnis zur Verbesserung der Kommunikations- und Informationstechnologie in der Intensiv- und Notfallmedizin; [38]) entwickelt, die verteilte Datenausleitungsprozesse realisieren. Somit konnten innerhalb der MII bspw. unterschiedliche Middlewares (AKTIN und DSF) für die Anbindung an das Deutsche Forschungsdatenportal für Gesundheit (FDPG) genutzt werden, um automatisierte, föderierte Machbarkeitsanfragen auf den KDS-konformen Datensätzen der DIZ auszuführen. Um die Komplexität der Gesamtinfrastruktur und der administrativen Aufwände zu reduzieren, wird das DSF als Use-Case-unabhängige verteilte Business Process Engine seit 2024 unterstützt, da es parallel verschiedene Prozesse ausführen kann [36, 37].

Außerdem wurden in der TF ÜS zur Anbindung der DIZ an die zentralen Dienste der MII harmonisierte Schnittstellenspezifikationen erarbeitet, etwa für Machbarkeitsanfragen und das Antrags- und Vertragsmanagement im FDPG. Weitere Beschreibungen, wie z. B. für zentrale Ausleitungsprozesse mit pseudonymisierten Daten und für verteilte Analysen, sind derzeit in Arbeit.

Auf Basis der Konzepte und Spezifikationen wurden nationale, dezentrale Test- und Produktiv-Infrastrukturen etabliert. Interoperable Machbarkeitsanfragen, zentrale Datenausleitungsprozesse und verteilte Analysen werden auf dieser Infrastruktur durchgeführt und kontinuierlich weiterentwickelt.

Für Erprobungen in der Testinfrastruktur ist das Vorhandensein von Testdaten entscheidend, die klinisch relevante Elemente aus den KDS-Strukturen abdecken müssen. Die TF DDT stellt Werkzeuge für die Generierung synthetischer Daten mit solchen Eigenschaften zusammen, um hohen Aufwänden der Testdatenerstellung zu begegnen [39–41]. Aufgrund der Freiheitsgrade der KDS-Spezifikationen ist es erforderlich, standortspezifische Testdaten zu generieren. Hierbei werden Notwendigkeiten zur Datenharmonisierung in den jeweiligen Datennutzungsprojekten ableitbar.

### Zentrale und verteilte Datenanalysen

In der MII erfolgt die Bereitstellung der Daten entweder zentral über DMSt mit pseudonymisierten Datensätzen unter Nutzung des Broad Consent (siehe auch Beitrag von Zenker et al. in diesem Themenheft) oder direkt über Verteilung lokal auszuführender Analyseskripte auf den dezentral verbleibenden Daten in den DIZ.

Die Bedienung von Datennutzungsprojekten benötigt zunächst geeignete Methoden der Selektion der betroffenen Patient:innen-Kohorte. Für die Selektion wurden je nach Verfügbarkeit verschiedene Verfahren etabliert, u. a. FHIR Search [42] und HL7 Clinical Quality Language [43]. Zur Aufbereitung der selektierten und bereitgestellten FHIR-Daten für fachspezifische Analysen durch die Forscher:innen (u. a. mit SPSS [IBM Corporation, Armonk, NY, USA] oder R [The R Foundation, Wien, Österreich]) wurden neue Werkzeuge wie „fhircrackr“ entwickelt [44].

Die TF VA entwickelte Vorgehensmodelle und Vorgaben für verteilte Analysen, die einen wichtigen Mechanismus zur Datennutzung in der MII darstellen. Bisher werden mehr als 80 % der im Transparenzportal des FDPG dargestellten Auswertungsprojekte verteilt durchgeführt. Dies stellt besondere Anforderungen an die Interoperabilität, insbesondere auf Prozess- und Schnittstellenebene. Zurzeit werden hauptsächlich projektspezifische Skripte zur Ausführung auf den DIZ-Datenbeständen genutzt. Die weitverbreitete Open-Source-Software DataSHIELD [45] wird ebenfalls für die MII pilotiert. Parallel wird im Projekt PrivateAIM die Entwicklung einer umfangreichen Plattform initiiert, die spezifisch auf die Anforderungen der MII ausgerichtet ist [46].

## Diskussion

Mit der interoperablen Infrastruktur hat die MII eine zukunftsweisende Lösung für den Austausch und die Nutzung von Routinedaten für die biomedizinische Forschung vorgestellt. Die gewählten Ansätze werden nicht nur in der MII mit ihren klinischen Use Cases, Methodenplattformen und Fortschrittshubs (siehe auch Beitrag von Krefting et al. in diesem Themenheft) verwendet, sondern in Teilen auch durch weitere nationale Forschungsdatenverbünde, wie u. a. das Netzwerk Universitätsmedizin (NUM), die Nationale Forschungsdateninfrastruktur für Gesundheit und die German Biobank Alliance. Der Austausch mit vergleichbaren Initiativen wie SPHN (Swiss Personalized Health Network) in der Schweiz [47] und Health-RI in den Niederlanden [48] sowie die Kooperation mit nationalen und internationalen Akteuren im Umfeld der Standardisierung wie dem Interop Council, EHDS und HL7 ermöglichen eine kontinuierliche und gemeinsame Weiterentwicklung (siehe auch Beitrag von Waltemath et al. in diesem Themenheft).

Durch verschiedene gesetzliche Vorgaben in Deutschland und in der EU wird die formale und einheitliche Abbildung von vielen Bereichen der Gesundheitsversorgung im Standard HL7 FHIR zum festen Bestandteil der digitalen Transformation der Gesundheitssysteme. Hierdurch bestätigt sich die in der AG IOP gewählte Methode, FHIR als Informations- und Kommunikationsstandard einzusetzen, z. B. für die Inhalte des KDS und für die Austauschprozesse mit dem DSF. In Zukunft wird die Forschung mit Routinedaten von der Nutzung von FHIR in der Krankenversorgung massiv profitieren und die ETL-Implementierungsarbeit in den DIZ wesentlich erleichtern.

Die hohe Komplexität dieser Arbeiten ist besonders dort unvermeidbar, wo aufgrund großer Heterogenität der Systemlandschaft Interoperabilität noch hergestellt werden muss. Die Umsetzung von KDS-Modulen an den Standorten kann die Freiheitsgrade der Profilierung nutzen und zu unterschiedlichen Implementierungen führen. Vorgaben zur Interoperabilität von Real World Data im Gesundheitswesen implizieren nicht automatisch die vollständige Harmonisierung dieser Daten (z. B. in unterschiedlichen Einheiten gemessene Laborwerte). Dieser Diskrepanz kann durch die Verwendung von konkretisierenden IGs mit Subsets von FHIR-Profilen, Metadaten mit Provenienz sowie Testdaten für jedes Nutzungsszenario begegnet werden. Umfang und Ausprägung der Metadatenvokabulare müssen ihre praktische Eignung noch durch Implementierung und Nutzung nachweisen.

Da sich die Gesamtinfrastruktur noch im Aufbau befindet, sind derzeit noch viele manuelle Arbeitsschritte vor allem in den DIZ erforderlich. Aus dem Anspruch einer zukünftig besseren Automatisierung ergeben sich hohe Erwartungen hinsichtlich Umfang, Skalierung und Geschwindigkeit der Ausführung der Prozesse auf der verteilten Infrastruktur. Sowohl für zentrale Datenbereitstellungen als auch für verteilte Analysemethoden stehen dabei Schnittstellen und Prozesse im Fokus, die unabhängig von der jeweiligen Implementierung sind. Um der Heterogenität an den Standorten begegnen zu können, werden keine Vorgaben zum Einsatz bestimmter Softwarekomponenten gemacht. Für die Realisierung der übergreifenden Use Cases und Methodenplattformen entsteht außerdem ein großer Bedarf an weiterführenden Schnittstellenbeschreibungen, automatisierten Integrationstests und Testdaten.

Das Projektmanagement über alle MII-Teilprojekte und derzeit über 55 Organisationen hinweg ist anspruchsvoll und wird durch die gemeinsame Governance unterstützt. Durch die Anbindung vieler teilnehmender Institutionen an die verteilte Infrastruktur entstanden in der MII hohe Aufwände, die sich mit dem Hinzukommen weiterer Standorte und Datenquellen künftig noch vergrößern werden. Schulungen wie die MIRACUM-DIFUTURE-School sowie der Einsatz von Hackathons und Projectathons haben sich dabei für die Umsetzung als sehr hilfreich erwiesen.

Die Zusammenarbeit in der AG IOP und ihren Taskforces ist entscheidend, um Schwierigkeiten zu beheben und Harmonisierung herbeizuführen. Insbesondere für die Weiterentwicklung besteht hoher Ressourcenbedarf, wobei u. a. FHIR-Entwickler:innen, Terminologie-Expert:innen und engagierte Kliniker:innen benötigt werden. Für den nachhaltigen Fortbestand der technischen Infrastruktur wird auf Open-Source-Entwicklungen gesetzt. Die DIZ werden von vornherein einbezogen, um die lokale Umsetzung zu ermöglichen und die Anforderungen an die verteilte Infrastruktur zu berücksichtigen. So wird in der MII eine nachhaltige Community aufgebaut, welche die Arbeiten kommuniziert, koordiniert und priorisiert.

In der AG IOP wurden folgende offene Themen identifiziert, für die eine vollständige Lösung noch erarbeitet wird:

- die Verstetigung der erfolgreich etablierten Arbeitsstrukturen der MII in Zusammenarbeit mit dem NUM,
- die Integration eines nationalen Terminologieservers,
- die Kuratierung der Datenqualität an unterschiedlichen Stellen u. a. bereits in der Primärdokumentation,
- die Harmonisierung der Daten für die wissenschaftliche Auswertbarkeit über reine Interoperabilität hinaus,
- die Adressierung spezifischer, von der Krankenversorgung abweichender Anforderungen komplexer Abfragen und Analysen in der MII, z. B. zur Kohortenbildung,
- die nachhaltige Umsetzung verteilter Analysen durch geeignete Plattformen und sichere Vorgehensweisen,
- der Vergleich der gewählten Verfahren in der MII-Infrastruktur mit anderen Ansätzen, z. B. mit Common Data Models [49],
- die Ausbildung und Motivation kompetenter Mitarbeiter:innen und Fachexpert:innen für die aktive Mitarbeit in den MII-Gremien.

Diesen und weiteren Themen wird in den kommenden Jahren verstärkt Aufmerksamkeit gewidmet.

## Fazit

Der Aufbau der nationalen Infrastruktur zur Sekundärnutzung von Versorgungsdaten in der MII hat sich als sehr erfolgreich erwiesen, mit inzwischen mehr als 55 beteiligten Organisationen, rund 40 etablierten DIZ, 17 spezifizierten KDS-Modulen und über 500 Mio. in FHIR-Ressourcen bereitstellbaren Datensätzen. Die Definition von Informationsmodellen und ihre technische Umsetzung mittels FHIR und standardisierter Terminologien ermöglicht es trotz der hohen Komplexität und Heterogenität von Real World Data, Daten interoperabel auszutauschen und zu analysieren. Damit ist die MII gut gerüstet, um flexibel auf zukünftige Herausforderungen der technischen Integration und neue Forschungsfragestellungen zu reagieren und ihre Rolle in der digitalen Transformation des Gesundheitswesens weiter auszubauen.

## Footnotes

1 FHIR-Spezifikationen für die Interoperabilität nach § 373 SGB V.

2 FHIR-Spezifikationen für die elektronische Patientenakte ePA nach § 355 SGB V.
